# Different EPHX1 methylation levels in promoter area between carbamazepine-resistant epilepsy group and carbamazepine-sensitive epilepsy group in Chinese population

**DOI:** 10.1186/s12883-019-1308-4

**Published:** 2019-06-04

**Authors:** Yudan Lv, Xiangyu Zheng, Mingchao Shi, Zan Wang, Li Cui

**Affiliations:** Department of Neurology, The First hospital of Jilin University, 71-Xinmin Street, Changchun, People’s Republic of China

**Keywords:** Methylation, Bisulfite sequencing PCR, EPHX1,Northern Han Chinese

## Abstract

**Background:**

Epigenetics underlying refractory epilepsy is poorly understood. DNA methylation may affect gene expression in epilepsy patients without affecting DNA sequences. Herein, we investigated the association between Carbamazepine-resistant (CBZ-resistant) epilepsy and EPHX1 methylation in a northern Han Chinese population, and conducted an analysis of clinical risk factors for CBZ-resistant epilepsy.

**Methods:**

Seventy-five northern Han Chinese patients participated in this research. 25 cases were CBZ-resistant epilepsy, 25 cases were CBZ-sensitive epilepsy and the remaining 25 cases were controls. Using a CpG searcher was to make a prediction of CpG islands; bisulfite sequencing PCR (BSP) was applied to test the methylation of EPHX1. We then did statistical analysis between clinical parameters and EPHX1 methylation.

**Results:**

There was no difference between CBZ-resistant patients, CBZ-sensitive patients and healthy controls in matched age and gender. However, a significant difference of methylation levels located in NC_000001.11 (225,806,929.....225807108) of the EPHX1 promoter was found in CBZ-resistant patients, which was much higher than CBZ-sensitive and controls. Additionally, there was a significant positive correlation between seizure frequency, disease course and EPHX1 methylation in CBZ-resistant group.

**Conclusion:**

Methylation levels in EPHX1 promoter associated with CBZ-resistant epilepsy significantly. EPHX1 methylation may be the potential marker for CBZ resistance prior to the CBZ therapy and potential target for treatments.

## Background

According to international League Against Epilepsy (ILAE), about 65 millions of epilepsy people have been reported in the world [1], and approximately 36% of epilepsy patients have poor or no drug-response [2]. Epigenetic modification means genomic reprogramming without affecting DNA sequence, such as microRNA expression, histone modification, and especially DNA methylation [3, 4]. The most common DNA methylation usually happens at 5’carbon of cytosine in CpG islands within the promoters [5]. Additionally, in recent study, DNA methylation has been suspected as one of the main epigenetic mechanisms in epilepsy and DNA methylation may affect gene expression in epilepsy without affecting DNA sequences [6, 7].

Carbamazepine (CBZ) known as a first-line anticonvulsants, was commonly used for the treatment of partial seizures. However, about 30–40% of epilepsy patients were CBZ-resistant according to the recent studies [8, 9]. Among Cytochrome P450 proteins (CYP), the CYP3A4 or CYP2C8 palys an important role in the the metabolization of CBZ or the formation of carbamazepine 10–11 epoxide (CBZ-E), and CBZ-E has been known as an active CBZ metabolite equipotent [10, 11]. In addition to CYP3A4 and CYP2C8, Microsomal Epoxide Hydrolase (EPHX1) also participated in the biotransformation of CBZ as major drug-metabolizing enzymes. EPHX1 is responsible for the conversion of the inactive water-soluble metabolite CBZ 10,11-diol from CBZ 10,11-epoxide [12]. ATP Binding Cassette Subfamily B Member 1 (BABCB1) and ATP Binding Cassette Subfamily C Member 2 (ABCC2) have been implicated in transport of CBZ 10–11 epoxide [13, 14]. Additionally, EPHX1 has significant biochemical effect on the carbamazepine-diol/carbamazepine-epoxide ratios, which may increase the effective theraphy of CBZ and decrease the adverse effects.

Herein, we investigated the association between CBZ-resistant epilepsy, CBZ-sensitive epilepsy and EPHX1 methylation in a northern Han Chinese population, and conducted an analysis of clinical risk factors for CBZ-resistant epilepsy. The goal of the study was to determine differential methylation profiles of EPHX1-candidate gene in CBZ-resistant epilepsy.

## Methods

### Patients

All of the enrolled subjects (patients and normal controls) were of Northern Han Chinese ethnicity. Patients were from five towns in Jilin Province (Hua Dian, Pan Shi, Tao Nan, Jiao He, and Shu Lan). The patients have provided written informed consent and this research has been approved by the First Hospital of Jilin University’s Research Ethics Board.

During the years 2014–2016, 25 cases that fulfilled the diagnostic criteria for CBZ-resistant epilepsy were identified at the Department of Neurology, First Hospital of Jilin University, Changchun, China. CBZ-resistant epilepsy was defined as: 1 CBZ-monotheraphy for epilepsy; 2 total dose per day was more than 1.0 g or plasma concentration more than 10 μg/ml; 3 invalid theraphy without any change in seizure frequency. CBZ-sensitive epilepsy was defined as: 1 CBZ-monotheraphy for epilepsy; 2 total dose per day was less than 0.8 g or plasma concentration less than 10 μg/ml; 3 significant decrease in seizure frequency (≥70%). In addition, normal control group involved 25 persons.

Data collected from the patients’ records included: age, gender, final dose of CBZ, seizure frequency and disease course.

## Method

### Methylation prediction

Using a CpG island searcher (www.softberry.com/berry.phtml?topic=cpgfinder&group=programs&subgroup=promoter), we found that EPHX1 possess obvious CpG islands in their promoter regions.

### DNA extraction

DNA was extracted from peripheral blood using a SK8224 blood mini kit (Sangon Biotech, Shang Hai, China). We use methprimer to make the forward and reverse primers. The sequences for EPHX1 were M247-F (5′-TGTGGTGGAATGATATTAGTTAAGGT-3′) and M247-R (5′-ACCACATTCCCTAACTTCAACTACA-3′).

### Bisulfite sequencing PCR (BSP)

To verify the methylation level of genomic DNA from the peripheral blood, BSP was used. DNA was first modified by treatment with sodium bisulfite to convert all ‘C’s to uracil residues except 5 mCs. Then bisulfite-modified DNA were amplified by PCR, which performed in a RT-PCR instrument (Verity 96well, ABI, USA) using 2 × Power Taq PCR MasterMix (Sangon Biotech, Shang Hai, China) under the Touch-down program: 98 °C for 4 mins, followed by 9 cycles of 94 °C for 45 s, 66 °C for 45 s (decrease 1 °C per cycle), 72 °C for 1 min, then followed by 40 cycles of 94 °C for 45 s, 56 °C for 45 s, 72 °C for 1 min, and final extension 72 °C for 8 mins. Moreover, the PCR product was recovered by TIANgel Midi Purification Kit (Sangon Biotech, Shang Hai, China) after verification in a 2% agarose gel. Then the purified DNA was ligated into the vector pUC18-T by pUC18-T Cloning Kit with Competent Cell (Sangon Biotech, Shang Hai, China) and transformed into *E. coli* strain TOP10. Sequence determinations were carried out at SANGON (Shanghai, China) [15].

### Statistical analysis

BSP sequences data are analyzed using software from BiQ Analyzer (http://biq-analyzer.bioinf.mpi-inf.mpg.de/) to calculate the level of DNA methylation (methylation level = methylated CpG dinucleotides/total CpG dinucleotides). Statistical analysis was carried out by Prism7.0 using ANOVA to determine statistical significance in EPHX1 methylation levels between different groups (the criteria for significance were defined as *p* < 0.05). Quantitative data is expressed as mean ± SD and analyzed by one-way ANOVA. Comparison between the groups was made by analyzing data with post-hoc method (Tukey). Statistical significance was set at a level of *P* < 0.05. And Pearson’s correlation was performed to determine the association between EPHX1 methylation and seizure frequency, disease course (the criteria for significance were defined as *P* = 0.01 or *P* = 0.05).

## Results

### Clinical parameter evaluation

The clinical characteristics, EPHX1 methylation distribution between different groups were shown in Tables 1 and 2 respectively. Clinical Parameters included the age, gender, final dose of CBZ, seizure frequency and disease course.

**Table 1:** Clinical characteristics and EHPX1 methylation in the 25 patients with CBZ-resistant epilepsy

ID No.	Gender	Age	Final-dose (mg)	CBZ-concentration in blood (ug/ml)	CBZ-concentration / Final dose (Ratio)	Seizure-frequency	Course of disease (year)	EHPX1 methylation
1	M	40	1000	5.6	5.6/1000	2/week(96/year)	7	25%
2	F	43	1000	6.5	6.5/1000	3/week(144/year)	11	29%
3	F	47	1000	4.8	4.8/1000	3.5/week(168/year)	4	31%
4	M	38	1000	5.2	5.2/1000	3/week(144/year)	8	30%
5	F	40	1000	6.7	6.7/1000	2/week(96/year)	7	22%
6	M	39	1000	7.2	7.2/1000	1.5/week(72/year)	4	21%
7	F	43	1200	6.8	5.7/1000	5/week(240/year)	3	54%
8	M	51	1000	7.6	7.6/1000	4/week(192/year)	5	33%
9	F	55	1200	6.2	5.1/1000	3.5/week(168/year)	10	53%
10	F	38	1000	8.1	8.1/1000	3/week(144/year)	6	26%
11	F	40	1000	7.3	7.3/1000	4/week(192/year)	3	31%
12	F	42	1000	6.6	6.6/1000	2.5/week(120/year)	4	24%
13	M	51	1200	7.2	6.0/1000	4.5/week(216/year)	7	48%
14	F	37	1000	6.5	6.5/1000	3/week(144/year)	6	41%
15	M	45	1000	6.1	6.1/1000	2.5/week(120/year)	4	51%
16	M	41	1000	7.2	7.2/1000	3/week(144/year)	6	30%
17	M	37	1200	4.7	3.9/1000	3.5/week(168/year)	5	50%
18	F	40	1000	7.7	7.7/1000	3/week(144/year)	2	30%
19	F	35	1000	8.4	8.4/1000	4/week(192/year)	3	26%
20	F	36	1000	7.8	7.8/1000	4/week(192/year)	6	38%
21	F	40	1000	8.9	8.9/1000	3/week(144/year)	9	24%
22	M	47	1200	9.2	7.6/1000	4.5/week(216/year)	17	26%
23	M	50	1000	6.9	6.9/1000	3/week(144/year)	12	46%
24	F	46	1000	6.4	6.4/1000	4/week(192/year)	11	48%
25	M	32	1000	5.8	5.8/1000	4/week(192/year)	8	57%

**Table 2:** Clinical characteristics and EHPX1 methylation in the 25 patients with CBZ-sensitive epilepsy

ID No.	Gender	Age	Final-dose (mg)	CBZ-concentration in blood (ug/ml)	CBZ-concentration / Final dose (Ratio)	Seizure-frequency	Course of disease (year)	EHPX1 methylation
1	M	45	600	8.3	14.1/1000	1–2/year	4	32%
2	F	38	400	4.1	10.2/1000	2–3/year	5	6%
3	F	46	700	6.4	8.9/1000	1/year	3	28%
4	M	41	600	7.2	12.2/1000	–	4	25%
5	M	35	800	6.8	8.5/1000	–	4	27%
6	M	37	200	2.6	13.0/1000	2–3/year	5	24%
7	F	40	600	5.7	9.7/1000	1–2/year	6	23%
8	F	43	600	5.3	9.0/1000	–	4	16%
9	M	48	800	7.2	9.0/1000	1–2/year	5	12%
10	M	39	600	5.6	9.5/1000	–	2	27%
11	F	43	600	5.2	10.9/1000	–	2	32%
12	F	50	700	6.4	8.9/1000	1–2/year	3	24%
13	F	47	600	5.7	9.7/1000	1–2/year	1	11%
14	M	52	600	6.4	10.9/1000	–	3	13%
15	F	33	800	7.5	9.4/1000	3–4/year	4	17%
16	M	54	700	7.1	9.9/1000	2–3/year	2	20%
17	M	36	600	6.7	11.4/1000	–	3	24%
18	M	38	600	6.8	11.6/1000	–	2	28%
19	F	40	600	5.5	9.4/1000	1–2/year	6	16%
20	M	42	400	4.6	11.5/1000	–	4	30%
21	F	44	600	5.5	9.4/1000	2–3/year	3	26%
22	M	48	400	4.7	11.8/1000	–	4	22%
23	F	39	400	4.9	12.3/1000	–	3	17%
24	M	36	700	6.0	8.4/1000	1–2/year	3	20%
25	F	47	600	5.9	10.0/1000	1–2/year	2	14%

There was no significant difference between CBZ-resistant epilepsy patients, CBZ-sensitive epilepsy patients and healthy controls in terms of age (42.12 ± 1.132, 42.44 ± 1.112 versus 42.68 ± 1.727, *P = 0.3938, P = 0.4538*), gender (male/female: 11/14, 13/12 versus 12/13). The final dose of CBZ in resistant-group was 1000 mg/day or 1200 mg/day, and the final dose in sensitive-group was changed from 200 mg/day to 800 mg/day. CBZ-resistant epilepsy patients has longer disease course than CBZ-sensitive epilepsy patients (6.720 ± 0.6965 versus 3.480 ± 0.259, *P<0.01*).

Furthermore, we defined the ratio of CBZ-concentration in blood and CBZ-final dose as N/1000, such as 5.6/1000, 7.1/1000. Then we compared the ratio between CBZ-resistant group and CBZ-sensitive group, there was a significant difference (6.624 ± 0.2401 versus 10.38 ± 0.3019, *P<0.001*). Such results indicated that CBZ-resistant group has a much lower CBZ-metabolites concentration than CBZ-sensitive group.

### Methylation in CBZ-resistant epilepsy group and CBZ-sensitive epilepsy group

**Fig. 1:**
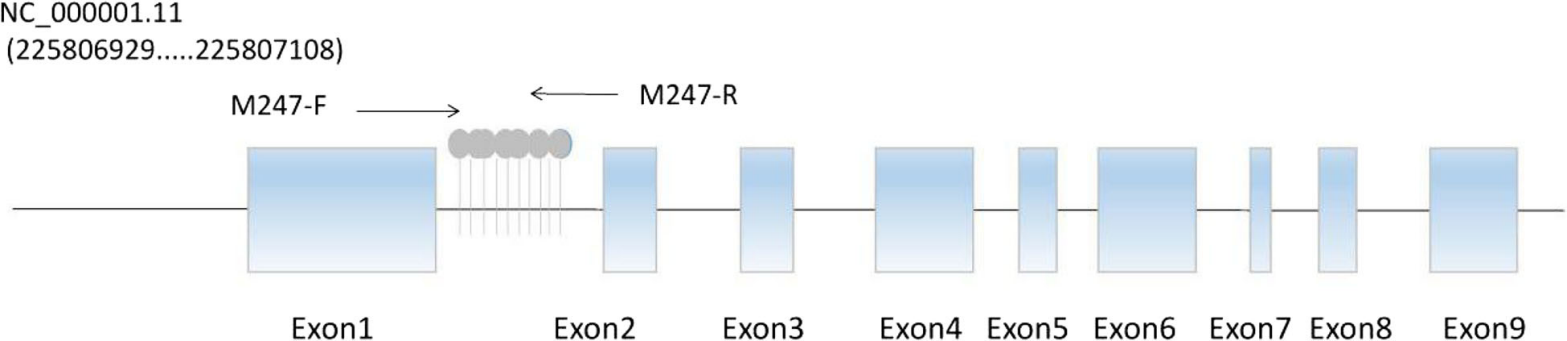
The schematic diagram of the CpG sites in the EPHX1 promoter. CpG sites are depicted by lollipop markers.forward and reverse primers are shown as arrows

**Fig. 2:**
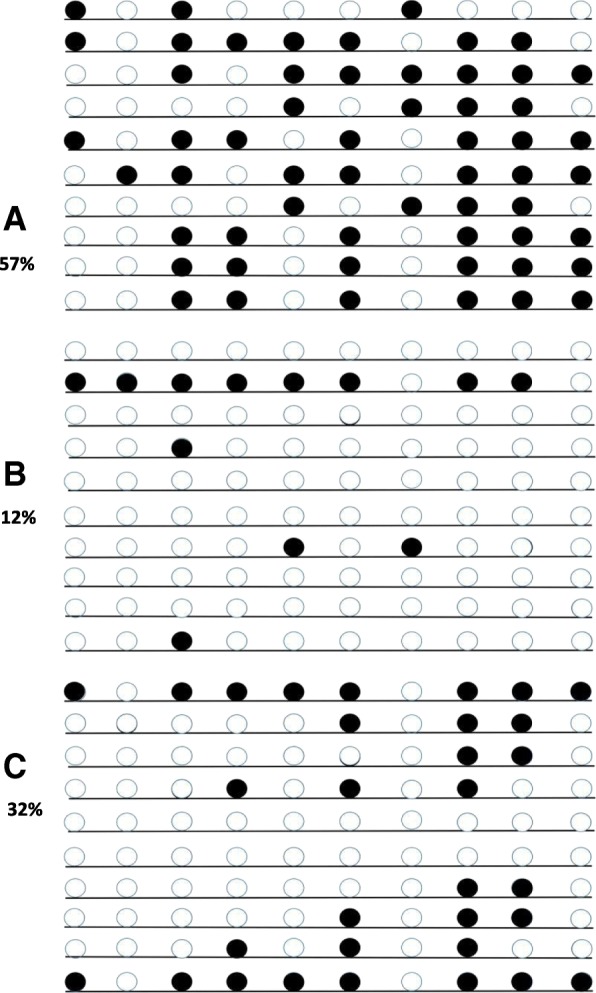
EPHX1 methylation density between CBZ-resistant group, CBZ-sensitive group and controls. **a** CBZ-resistant group, **b** control group, **c** CBZ-sensitive group. White cycle: unmethylated CpG dinucleotide; Black cycle: methylated CpG dinucleotid

**Fig. 3:**
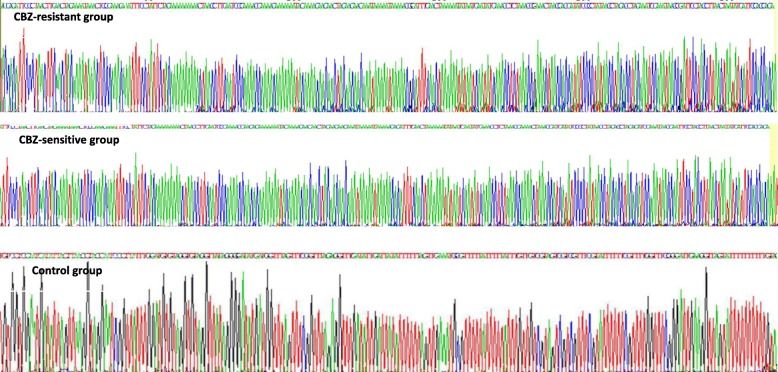
EPHX1 methylation density in promoter between CBZ-resistant group, CBZ-sensitive group and controls (Blue lines represents the methylation location)

For the EPHX1, we used CpG island searcher to predict the CpG clusters and found that there was a large CpG clusters located in the reference sequence NC_000001.11 (225,806,929.....225807108), which contained 10 CpG sites and located in promoter before extron1 as seen in Fig. 1. As shown in Fig. 2, among the 10 sequence fragments, the maximium methylation level of the CpG sites was 12% in controls, 57% in CBZ-resistant patients, and 32% in CBZ-sensitive patients. Two healthy controls had no EPHX1 methylation. In addition, methylation levels depend on CpG sites in the EPHX1 promoter. And different distribution of CG-base density among CBZ-resistant group, CBZ-sensitive group and controls have been shown in Fig. 3.

**Fig. 4:**
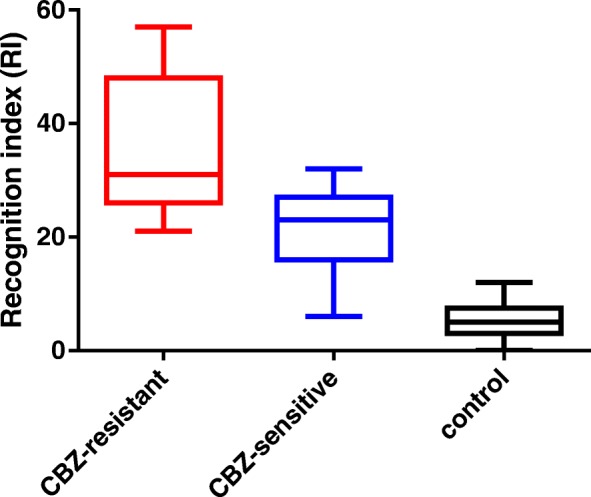
Different methylation distributions between CBZ-resistant group, CBZ-sensitive group and controls

Besides this, different distributions of methylation levels between CBZ-resistant group, CBZ-sensitive group and controls have been presented in Fig. 4. Such results indicated that both CBZ-resistant group and CBZ-sensitive group have much higher methylation levels than controls, and the methylation level in CBZ-resistant group was higher than CBZ-sensitive group.

### Association between EPHX1 methylation and seizure frequency, disease course

As shown in Table 1, the clinical characteristics and EPHX1 methylation of the CBZ-resistant epilepsy patients have been described. We used Pearson’s correlation to make an analysis of the association between EPHX1 methylation and age, final dose, seizure frequency, disease course and ratio of CBZ-concentration and CBZ-final dose. And as shown in Table 3, in CBZ-resistant group, there was a significant correlation found between seizure frequecy, ratio and EPHX1 methylation (*r* = 0.4921/*P* = 0.0125,-0.5289/0.0066 respectively), and no significant correlation was found between age, disease course and EPHX1 methylation (*r* = 0.2382/*P* = 0.2515, *r* = 0.06833/*P* = 0.7455, respectively).

**Table 3:** Correlation between Age,frequency, disease course, Ratio and EHPX1 methylation in different groups

	*r值*		*P值*	
	EHPX1methylation In CBZ-resistant group	EHPX1methylation In CBZ-sensitive group	EHPX1methylation In CBZ-resistant group	EHPX1methylation In CBZ-sensitive group
Age	0.2382	−0.1429	0.2515	0.4955
Frequency	0.4921*	−0.3498	0.0125*	0.0865
Disease course	0.06833	−0.1354	0.7455	0.5188
Ratio (CBZ-concentration / CBZ-dose)	−0.5289**	0.3095	0.0066**	0.1322

There is a significant correlation between frequency, ratio and EHPX1-methylation in CBZ-resistant group (P*<0.05, P**<0.01)

Additionally, in CBZ-sensitive group as shown in Table 3, there is a correlation was found between seizure frequecy and EPHX1 methylation (*r* = − 0.3498/*P* = 0.0865), but no statistical significance.

## Discussion

We found a significant high EPHX1 methylation level in CBZ-resistant group in northern Han Chinese epilepsy patients. Previous studies focused on the EPHX1 polymorphisms in refractory epilepsy patients, few discussed the EPHX1 methylation.

As we known, about one third of the epilepsy population have poor response or no response to the antiepileptic therapy [16, 17]. And studies show that the variability in drug-resistance not only comes from the environmental factors, but also associates with genetic background and heterogeneity [18]. Genetic background such as genetic polymorphisms or genetic methylation can predict drug individually and efficiently. Makmor-Bakry et al. demonstrated that the maintenance dose of CBZ associated with EPHX1 416A.G and 337C.T polymorphisms. Moreover, a higher diol/epoxide ratio associated with 377C.T, and a lower ratio associated with 416A.G polymorphism [19]. But, there was conflicting reports in the literature, such as that Antonietta caruso demonstrated that EPHX1 and CYP3A4 polymorphisms have no effect on CBZ 10,11-epoxide levels [20]. So, under such situation, it was necessary to evaluate effects of EPHX1 methylation on CBZ-metabolism.

Additionally, EPHX1 has been known as a biotransformation enzyme localized mainly in the endoplasmic reticulum of eukaryotic cells. It has two types, namely microsomal EPHX1 (OMIM: 132810) and soluble EPHX2 (reviewed by Harris and Hammock [21]). EPHX1 consists of nine exons and encodes three transcription variants, which was first shown to convert epoxides such as styrene oxide, 1-methyl-1-phenyloxirane, indene 1,2-oxide, and cyclohexene oxide into the respective diols [22]. As described above, the EPHX1 seems to show a detoxifying function. Besides this, in many areas of the brain, such as frontal or occipital lobe, pons, red nucleus, and cerebellum, the EPHX1 transcripts have been found. Due to its biochemical effect on the carbamazepine-diol/carbamazepine-epoxide ratios, EPHX1 has been studied as a targed pharmacological point to increase the effective theraphy of anti-epileptic drugs and decrease the adverse effects. However, there are conflicting results have been found between the EPHX1 gene variability and anti-epileptic drugs concentration. Subsequently, studies on the EPHX1 methylation may be another choice to explore the potential association.

By sequence analysis, we found that much more CG bases in promoter area in carbamazepine-resistant epilepsy patients or carbamazepine-sensitive epilepsy patients. However, in the following study, our results demonstrated that the methylation levels of such CpG sites in CBZ-resistant group were much higher than carbamazepine-sensitive group or healthy controls (*P* < 0.001). As the result was shown above, it means that high methylation levels in CBZ-resistant group may predict poor antiepileptic therapy. Additionally, the EPHX1 methylation positively correlated with seizure frequency and final doses in CBZ-resistant group,

which also indicated that EPHX1 methylation plays an important role in the CBZ resistance response.

In this study, we investigated EPHX1 methylation in the peripheral blood DNA and tested the association between CBZ-metabolites concentration. Methylation levels in peripheral blood DNA have been widely investigated in various diseases, including diabetes [23], obesity [24], major depression [25], and various cancers [26–28]. Here we provided its strong association with lower CBZ-metabolites concentration in CBZ-resistant group. Glossop et al. demonstrated that DNA methylation pattern vary dramatically between blood cell types and tissues [29]. In our study, tissues in the CBZ-resistant epilepsy patients were not available, but we may obtain the brain tissues from the drug-refractory patients after the epilepsy surgery in future and test our results furthermore. On the other hand, high EPHX1 methylation levels in peripheral blood DNA in CBZ-resistant epilepsy group was also meaningful equally.

## Conclusions

In summary, although CBZ is the first choice drug for several epilepsy types, genetic variation in CBZ metabolic pathway like EPHX1 methylation contributed, in part, to the differences in patients’ response. We found a significant association between EPHX1 methylation and CBZ-resistant epilepsy patients in the northern Han Chinese patients, and EPHX1 methylation may be the potential marker for CBZ resistance prior to the CBZ therapy. It should be noted that our results need to be further confirmed by a future study with a larger sample size or tissues samples.

## Abbreviations

ABCB1: ATP Binding Cassette Subfamily B Member 1
ABCC2: ATP Binding Cassette Subfamily C Member 2
BSP: Bisulfite sequencing PCR
CBZ: Carbamazepine
CBZ-E: Carbamazepine 10–11 epoxide
CYP: Cytochrome P450 proteins
EPHX1: Microsomal Epoxide Hydrolase
ILAE: International League Against Epilepsy
